# Fas/FasL Contributes to HSV-1 Brain Infection and Neuroinflammation

**DOI:** 10.3389/fimmu.2021.714821

**Published:** 2021-08-30

**Authors:** Malgorzata Krzyzowska, Andrzej Kowalczyk, Katarzyna Skulska, Karolina Thörn, Kristina Eriksson

**Affiliations:** ^1^Department of Rheumatology and Inflammation Research, Sahlgrenska Academy, University of Gothenburg, Gothenburg, Sweden; ^2^Department of Virology and Cell Biology, Łukasiewicz Research Network - PORT Polish Center for Technology Development, Wroclaw, Poland; ^3^Laboratory of Nanobiology and Biomaterials, Military Institute of Hygiene and Epidemiology, Warsaw, Poland

**Keywords:** herpes simple type 1, neuroinflammation, Fas/FasL, microglia, chemokine, cytokines

## Abstract

The Fas/FasL pathway plays a key role in immune homeostasis and immune surveillance. In the central nervous system (CNS) Fas/FasL is involved in axonal outgrowth and adult neurogenesis. However, little is known about the role of the Fas/FasL pathway in herpes encephalitis. In this study, we used a neuropathogenic clinical strain of herpes simplex virus type 1 (HSV-1) to explore infection-induced inflammation and immune responses in the mouse brain and the role of Fas/FasL in antiviral CNS immunity. HSV-1 CNS infection induced the infiltration of Fas- FasL-bearing monocytes and T cells in the brain and also to an up-regulation of Fas and FasL expression on resident astrocytes and microglia within infected sites. Upon infection, Fas- and FasL-deficient mice (lpr and gld) were partially protected from encephalitis with a decreased morbidity and mortality compared to WT mice. Fas/FasL deficiency promoted cell-mediated immunity within the CNS. Fas receptor stimulation abrogated HSV-1 induced activation and inflammatory reactions in microglia from WT mice, while lack of Fas or FasL led to a more pronounced activation of monocytes and microglia and also to an enhanced differentiation of these cells into a pro-inflammatory M1 phenotype. Furthermore, the specific immune system was more efficient in Fas- and FasL-deficient mice with significantly higher numbers of infiltrating HSV-1-specific cytotoxic T cells in the brain. Our data indicate that the Fas/FasL pathway leads to excessive neuroinflammation during HSV-1 infection, which is associated with a diminished anti-viral response and an excessive neuroinflammation.

## Introduction

Human alphaherpesvirus 1 (HSV-1) infection affects approximately 60% to 95% of adults worldwide. HSV-1 causes infections in the form of oral lesion, and after primary infection, latency is established in sensory ganglia with a risk of recurring disease. Herpes simplex encephalitis (HSE) is the most common form of encephalitis constituting 10% to 20% of viral encephalitis cases (1, 2). Although all age groups are affected, its incidence is most common and severe in children and the elderly (2). HSE is associated with significant morbidity and mortality. More than 70% of patients without treatment die, and up to 30% despite anti-viral treatment. The majority of HSE patients suffer from epilepsy, mental retardation and/or chronic neuronal deficits, and only 2-5% of patients recover completely (2).

The Fas (CD95, APO-1)-signaling pathway is a common way to induce apoptosis in cells. Its physiological ligand, FasL (CD95L), belongs to the tumor necrosis factor (TNF) family. Fas and FasL play critical roles in the immune system, particularly in the killing of pathogen-infected target cells and the death of autoreactive lymphocytes (3). Although Fas is not normally expressed on central nervous system (CNS) cells, its expression can be induced within inflammatory sites in oligodendrocytes, resulting in their susceptibility to FasL-induced death (4, 5). Neuronal tumors can express Fas, but they are not susceptible to FasL-induced apoptosis (6). However, Fas-FasL activity causes infiltration of peripheral myeloid cells to the injury site in CNS resulting in inflammation-related neuronal cell death (7). Within the CNS, FasL expression is detectable in neurons, microglia and perivascular astrocytes (8). FasL promotes dendritic branching in immature neurons, outgrowth of dorsal root ganglions *in vitro*, ongoing and injury-induced neurogenesis in adult brains as well as migration of glioblastoma cells (6–12).

The involvement of FasL-mediated apoptosis in the resolution of viral infection in the CNS has been reported in West Nile virus (13) and mouse hepatitis virus (14) infections. Furthermore, olfactory receptor neurons prevent dissemination of neurovirulent influenza A virus into the brain by undergoing apoptosis due to up-regulation of FasL and activation of the JNK-stress pathway (15). On the contrary, Baloul et al. (16) had demonstrated that fatal rabies virus infection involved the early triggering of FasL production leading to the destruction of migratory T cells by the Fas/FasL apoptosis pathway.

We have previously shown that infection with human alphaherpesvirus 2 (HSV-2), a close related species of HSV-1 causing genital infection, leads to up-regulation of Fas and FasL expression by keratinocytes, but also renders cells resistant to Fas-induced apoptosis (17). Furthermore, monocytes from HSV-2 infected Fas (lpr) and FasL (gld)- deficient mice underwent delayed apoptosis and produced significantly less CXCL9, CXCL10 and TNF-α than monocytes in the wild type mice (17), which resulted in impaired recruitment of NK, CD4+ and CD8+ T cells within the infection sites followed by delayed virus clearance from the vaginal tissue and increased mortality (17).

We hypothesize that mounting of effective anti-HSV-1 defense within the nervous system depends on activation of the non-apoptotic Fas/FasL pathway. To test out hypothesis, we studied HSV-1 infection of the CNS using a well-established murine model in strains with mutation in Fas (lpr) or FasL (gld).

## Materials and Methods

### Virus

HSV-1 (strain ID 2762) isolated from a patient with herpesvirus encephalitis (kindly provided by professor Thomas Bergström, Department of Virology, University of Gothenburg) was grown and titrated in Vero cells (ATCC^®^ CCL-81) and kept at −80°C until use. Virus was diluted in phosphate-buffered saline (PBS) (pH 7.4) and maintained on ice until administered to mice maximum one hour later.

### Mice and Infection

Male mice, 6- to 8-week old, were used for all experiments. C57BL/6, B6. MRL-Fas lpr/J (Fas−) and B6Smn.C3-Fasl gld/J (FasL−) mice were purchased from the Charles River (Dortmund, Germany) and a breeding colony was maintained at the animal facility of the Department of Rheumatology and Inflammation Research, University of Gothenburg. Mice were kept under standard environmental conditions of temperature and light and were fed laboratory chow and water ad libitum. The experiments were approved by the Animal Research Ethical Committee of Gothenburg, and animal experimentation guidelines were strictly followed. Mice were anesthetized with isoflurane (Baxter, Lund, Sweden), and a total dose of 2 x 10^6^ PFU (plaque forming units) of HSV-1 in 20 μL was given per 10 μL into each nostril. Mice were monitored and scored according to the following scale: 0, no signs of infection/inflammation; 1, small nasal bump, watery eyes, jumpy behavior; 2, moderate nasal bump, conjunctivitis, ruffled hair; 3, large nasal bump, hunched/lethargic, severe conjunctivitis with swelling and hair loss, ruffled hair; 4, hunchback, severe conjunctivitis, weight loss above 20%, paralysis. Mice scoring 4 were immediately removed from the experiment, and subjected to isolation of brains and trigeminal ganglia.

### Quantitative PCR

Total DNA and RNA from the brain and trigeminal ganglia tissues were isolated at day 6, 7 and 8 post-HSV-1 infection fix using RNA/DNA Extracol kit (Eurx, Gdansk, Poland), according to the manufacturer’s instructions. Two hundred nanograms of DNA was used to detect HSV-1 by qPCR with primers and probe for the viral envelope glycoprotein (gB), as described by Namvar et al. (18) in ViiA 7 (Fast block) (Applied Biosystems, Carlsbad, CA, USA) with Fast Advanced Master Mix (Thermo Fisher Scientific, MA, USA). Standard curve analysis was based on Ct values and serial of 10-fold dilutions of the plasmid standard containing the gB gene with an initial concentration of 2.62 ×10^6^ HSV-1 genome copy numbers per reaction. A standard curve was included in each PCR run. Data are expressed as the HSV-1 copy number per ng of the total DNA in the tissue.

Approximately 0.5 μg of total RNA isolated from brains and trigeminal ganglia, as described above, was converted to cDNA using MLV Reverse transcriptase (Invitrogen, Thermofisher Scientific). Expression of lytic/latency related genes, namely immediate early gene ICP0 and ICP27, leaky-late gene, gB, and latency-associated transcript (LAT) was measured as described by Menendez et al. (19). Quantitative PCR of viral genes was performed with the GoTaq^®^ SYBR PCR System (Promega) according to the manufacturer’s protocols. HSV-1 LAT and lytic genes were normalized to the mean threshold cycle (CT) of β-actin housekeeping gene. Uninfected samples were assigned a CT value of 40 (designating background) to use the 2−ΔΔCT cycle threshold (2^−ΔΔCT^) method (20).

qPCR reactions for cytokines and chemokines were carried out using Fast Advanced Master Mix (ThermoFisher Scientific and TaqMan^®^ probes for the detection of IL-1β (Mm00434228_m1), IFN-γ (Mm01168134_m1), IFN-α2 (Mm00833961_s1), IFN-α4 (Mm00833969_s1), IFN-α9 (Mm00833983_s1), TNF-α (Mm00443258_m1), IL-6 (Mm00446190_m1), CCL-2 (Mm00441242_m1), CXCL1 (Mm04207460_m1), CXCL9 (Mm00434946_m1), CXCL10 (Mm00445235_m1), Fas (Mm04206620_m1), FasL (Mm00438864_m1) and GADPH (Mm99999915_g1), according to the manufacturer’s instructions using the qPCR instrument ViiA 7 (Fast block) (Applied Biosystems). Results were analysed with the 2−ΔΔCT cycle threshold (2^−ΔΔCT^) method (21), as above.

### Flow Cytometry Analysis

Single cell suspensions were generated at day 8 post infection from trigeminal ganglia and brain of HSV-1-infected mice. Tissues from uninfected mice were used as controls. Tissues were pressed through a 70 µm cell strainer and washed in PBS/2% FBS. Cell suspensions were pre-treated with the Fc receptors block - rat anti-CD16/32 antibody (2.4G2) (BD Biosciences) according to the manufacturer’s protocol. The following antibodies were used: anti-CD3-FITC (145-2C11, ThermoFisher Scientific), anti-CD4-PE or BV421 (clone RM4-5, BD Biosciences), anti-CD8-PE or BV421 (clone 53-6.7., BD Biosciences), anti- NK1.1-APC (clone PK136, BD Biosciences), anti-CD11b-PE (RB6-8C5, BD Biosciences), anti-CD192-BV421 (clone 475301, BD Biosciences), anti-Ly6C-APC-Cy7 (clone AL-21, BD Biosciences), anti-IBA-1-FITC (clone EPR16588, Abcam), anti-GFAP-Alexa Fluor^®^ 488 (clone GA5), anti-GFAP-Alexa Fluor^®^ 647 (clone 2.2B10, Thermofisher Scientific), anti-CD86-PE (clone GL1, BD Biosciences), anti-CD206-APC (clone MMR, ThermoFisher Scientific), anti-CD11b-FITC (clone M1/70) (BD Biosciences), anti-NOS2-APC (clone CXNFT, ThermoFisher Scientific), anti-Arg-1-PE (clone A1exF5, eBioscience™), anti-CD95-BV421 or Alexa Fluor^®^647 (clone Jo2, Becton Dickinson), anti-CD178-PE or BV421 (clone MFL3, Becton Dickinson), anti-caspase-3 Alexa Fluor^®^647 (clone C92-605) and annexin V-APC (Becton Dickinson). For intracellular staining, BD cytofix/cytoperm fixation/permeabilization kit was used according to manufacturer’s instructions. HSV-1 specific T cells were detected with SSIEFARL-PE tetramer (ProImmune). Stained cells were acquired using BD FacsLyric (BD Biosciences) and analyzed using FlowJo software (Tree Star, Ashland, OR, USA).

### Confocal Microscopy

Brains were fixed in 4% paraformaldehyde/PBS, then saturated with 30%/PBS sucrose, frozen in liquid nitrogen, and cut into 12 μm cryostat sections. Sections were washed with PBS and incubated overnight at 4°C with primary antibodies diluted in working solution (2% BSA, 0.1% saponin in PBS). When using mouse monoclonal antibody, Mouse on Mouse (M.O.M.^®^) blocking reagent kit (Vector Laboratories, Peterborough, United Kingdom) was applied for pre-incubation. Antibodies used included: rabbit polyclonal anti-HSV-1/2 (Dako, Agilent, Santa Clara, CA, USA), APC-conjugated rat monoclonal anti-CD11b (clone M1/70, BD Biosciences), polyclonal goat anti-IBA1 (ThermoFisher Scientific), anti-GFAP(clone 2A5, Abcam), anti-Fas-biotin (clone Jo2, Becton Dickinson), anti-FasL-biotin (MFL3, Becton Dickinson), anti-NeuN-Alexa Fluor^®^ 647 (clone EPR12763, Abcam), anti-NeuN-biotin (clone A60, Millipore), anti-MBP (clone 12, Abcam) and anti-caspase-3 Alexa Fluor^®^647 (clone C92-605, Becton Dickinson). Biotinylated Abs were detected with Alexa Fluor™ 555 Tyramide SuperBoost™ Kit (ThermoFisher Scientific), while other primary antibodies were detected using Alexa Fluor^®^ 555 or 647 anti-mouse, Alexa Fluor^®^ 488^®^ anti-rabbit and Alexa Fluor^®^ 555 or 647 anti-goat polyclonal antibodies (ThermoFisher Scientific). Apoptotic cells were also detected using In Situ Cell Death Detection Kit, Fluorescein (Roche, Merck) according to manufacturer’s instructions. After final washing in PBS, slides were closed in SlowFade™ Diamond Antifade Mountant with 4-6-diamidino-2-phenylindole (DAPI; ThermoFisher Scientific). Sections were imaged by confocal microscopy using a Zeiss Laser Scanning Inverted Microscope LSM-700 equipped with 40X/1.3 Oil NA objective and Black Zen software (Carl Zeiss) at the Center for Cellular Imaging of the University of Gothenburg.

### Primary Cultures

Primary cultures of mixed glial cultures were established from whole brains of neo-natal C57BL/6, MRL-Fas lpr/J and B6Smn.C3-Fasl gld/J mice. After isolation, blood vessels and meninges were carefully removed. Next, the brains were digested with 0.25% trypsin/Hanks balanced salt solution (Thermo Fisher Scientific) for 10 minutes (mixed glial cultures). After enzymatic digestion, pieces were washed (Hanks balanced salt solution; ThermoFisher Scientific), then triturated through pipettes with progressively smaller tip diameters. After sufficient trituration, cells for primary neuronal cultures were suspended in Dulbecco’s modified Eagle’s/F12 medium with GlutaMAX (DMEM/F12) supplemented with 10% FBS, 100 units/ml penicillin, 100 µg/ml streptomycin (Thermo Fisher Scientific). After 48h, 5 ng/ml of murine recombinant granulocyte and macrophage colony stimulating factor (GM-CSF) (Sigma-Aldrich, St. Luis, MO, USA) was added to medium. Cells were grown in poly-D-lysine (25 μg/ml; Sigma) coated plastic culture plates at standard conditions. After reaching confluence (2-3 weeks), mixed glial cultures were used to obtain microglia as described by Draheim et al. (20). Fas receptor was stimulated with recombinant mouse Fas Ligand/TNFSF6 (R&D Systems, Minneapolis, MN, USA) according to producer’s instructions. Microglia were stimulated with with poly(I:C) (50 μg/ml) (Sigma-Aldrich).

### Chemokine and Cytokine Analysis

Brains at 8 day post infection were collected in PBS with 0.1 mg/ml soybean trypsin inhibitor (Sigma), 1.5 mM Pefabloc (Roche), 50 mM EDTA, and 0.1% bovine serum albumin and frozen at -20°C. After thawing, samples were permeabilized using 2% saponin over night at 4°C, and the supernatant was subsequently collected by centrifugation. Chemokine and cytokine analysis was performed using a mouse magnetic Luminex assay from R&D Systems for 23 cytokines/chemokines. Significant differences in comparison to control were detected for CCL2 [lowest level of quantification (LLOQ) = 51 pg/ml], CCL3 (LLOQ = 77 pg/ml), CCL4 (LLOQ = 75 pg/ml), CCL5 (LLOQ = 55 pg/ml), CXCL1 (LLOQ = 57 pg/ml), IFN-γ (LLOQ = 34 pg/ml), IL-6 (LLOQ = 38 pg/ml), IL-12 (LLOQ = 76 pg/ml), IL-13 (LLOQ = 37 pg/ml) according to manufacturer’s instructions and analyzed using Bio-Plex 200 system (Bio-Rad). The levels of CXCL9, CXCL10 and IFN-alpha were measured using Mouse CXCL9/MIG Quantikine ELISA Kit (R&D, Minneapolis, MN, USA, LLOQ 7.8 pg/ml), Mouse CXCL10/IP-10/CRG-2 DuoSet ELISA kit (R&D, LLOQ 62 pg/ml) and Mouse IFN-α ELISA Kit (R&D, LLOQ 12.5 pg/ml) according to manufacturer’s instruction.

### Statistics

For statistical analysis, GraphPad Prism version 7 (GraphPad software) were used. To compare the differences between the groups, the Mann–Whitney U test and Wilcoxon test, were used and the results are reported as mean ± standard error of the mean (SEM) unless indicated otherwise. The p < 0.05 was considered statistically significant.

## Results

### HSV-1 Infection Induce the Expression of Fas And FasL in Brains and Trigeminal Ganglia

Because prior studies had suggested a role for Fas-FasL dependent non-apoptotic signalling in herpes virus infection (17, 22), we hypothesized that HSV-1 infection may induce Fas and FasL expression within infection sites on different immune cell types and glial/neuronal cells. Although Fas is normally expressed at low levels, if at all, on neurons, during physiologic stress its expression can be induced (6, 11, 23). Similarly, although FasL expression is detected in microglia, neurons and perivascular astrocytes, virus infection may lead to its up-regulation (13–15).

To assess a potential effect of HSV-1 infection on the Fas and FasL expression in CNS, C57BL/6 mice were inoculated intranasally with the HSV-1 strain isolated from a patient with HSE and followed for 8 days. At day 3 post infection, we observed a decrease of the animal weight, followed by symptoms such as nasal bumps, conjunctivitis, hunched posture and progressing lethargy. To determine which regions of the brain that are infected with HSV-1 during primary infection, brain tissue was dissected and assayed for virus titers with qPCR (**Supplementary Figure 1**). Nervous tissue from moribund mice was harvested beginning at 6 days post infection (d p.i.) through 8 d p.i. At 7 day p.i. (d p.i.), the highest viral loads were found in cerebral hemispheres, midbrain and pons (diencephalon) compared to olfactory bulb and cerebellum (**Supplementary Figure 1A**). At day 8, viral loads declined in all tested areas (**Supplementary Figure 1A**). Confocal analysis of HSV-1 positive cells in the respective areas showed presence of HSV-1+ neurons in olfactory bulb, cerebellum, cortex and midbrain, followed by HSV-1+ astrocytes in subventricular areas, olfactory bulb and HSV-1+ microglia identified in all tested areas (**Supplementary Figure 1B**).

**Figure 1:**
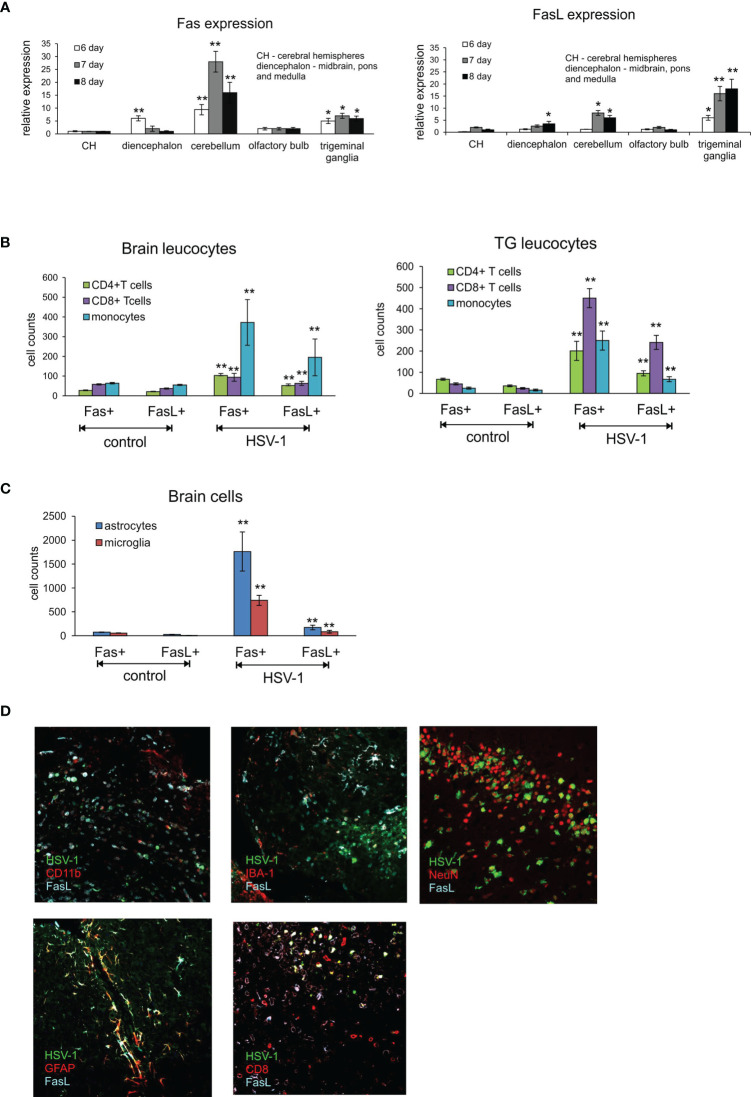
Fas/FasL expression correlates with HSV-1 infection in C57BL/6 mice. **(A)** Fas (left) and FasL (right) mRNA expressions in different brain parts: CH – cerebral hemispheres, diencephalon (midbrain, brainstem and medulla), cerebellum, olfactory bulb and trigeminal ganglia at 6-8 d p.i. were measured by quantitative real-time PCR. N = 10 animals at each time point. Data were presented as mean ± SEM. Data analysis was performed by comparing fold expression to uninfected control. **p ≤ 0.001, *p ≤ 0.05. **(B)** Leucocytes in brain (left) and trigeminal ganglia (right) homogenates expressing Fas or FasL were determined by flow cytometry at 8 d p.i. Data were presented as mean ± SEM. N = 7-9 animals. **Indicates p ≤ 0.001, *p ≤ 0.05 compared to uninfected controls. **(C)** Expression of Fas and FasL on resident brain cells – microglia and astrocytes – at 8 d p.i. Data were presented as mean ± SEM. N = 7-9 animals. **Indicates p ≤ 0.001, *p ≤ 0.05 compared to uninfected controls. **(D)** FasL expression on different cells in HSV-1 infected brains at 8 d p.i. Co-immunofluorescent staining for HSV-1 antigens (green), GFAP+ astrocytes (red), IBA-1 positive microglia (red), CD11b-positive monocytes (red), CD8+ T cells (red), neurons (NeuN, red) and FasL (turquoise). Midbrain (top left, middle and right, bottom right) and cortex (bottom left). Magnification x 200.

The highest expression of Fas mRNA in the infected brains was detected in cerebellum at 6, 7 and 8 d p.i. (**Figure 1A**) but also in cerebral hemispheres at 6 d p.i. (**Figure 1A**). Generally, Fas expression was up-regulated at 6 d p.i. to gradually decrease through day 8 (**Figure 1A**). In contrast, FasL expression was induced later, at 8 d p.i. and most significantly in cerebellum (**Figure 1A**). Trigeminal ganglia showed the highest up-regulation of FasL expression compared to brain, with the same tendency to increase later in infection (**Figure 1B**). Fas expression in trigeminal ganglia also increased at 7 and 8 d p.i. (**Figure 1A**). No Fas or FasL expression was detected on neurons.

T cells have crucial functions in controlling acute infection by some many neurotropic viruses (13, 14). In general, CD8+ T cells exert their antiviral functions *via* secretion of antiviral cytokines such as gamma interferon (IFN-γ) and and/or *via* a cytolytic pathway through the use of perforin-granzyme molecules or Fas-FasL interactions (24). However, also FasL-expressing myeloid cells could play a role during neuroinflammation (25). To determine if infection with HSV-1 favours the infiltration of immune cells bearing Fas and FasL into the CNS at the peak of primary infection, we performed flow cytometry analyses of the brain and trigeminal ganglia at day 8 p.i. and assessed the presence of Fas and FasL on CD4+ T cells (CD3+/CD4+ cells), CD8+ T cells (CD3+/CD8+ cells) and monocytes (CD45hi+/CD192+/CD11b+/Ly6C+ cells). As shown in **Figure 1B**, lymphoid cells expressing Fas and FasL infiltrated the brains of HSV-1-infected mice (**Figure 1B**). In contrast, in the trigeminal ganglia of HSV-1-infected mice, CD8+ T cells predominated and were the most abundant cell type bearing Fas and FasL (p ≤ 0.01) (**Figure 1B**), followed by CD4+ T cells and infiltrating monocytes (p ≤ 0.01) (**Figure 1B**).

Since also microglia and astrocytes can up-regulate Fas or FasL expression in response to viral infection or stress (23), we assessed the expression of Fas and FasL on astrocytes (GFAP+) and microglia cells (CD45low+/CD192-/IBA-1+). At 8 day p.i. we found significantly increased numbers of both Fas and FasL-expressing astrocytes and microglia (p ≤ 0.01) (**Figure 1C**). To further co-localise the presence of FasL-expressing cells with HSV-1 CNS infection, we stained the brain tissue at 8 d p.i. for astrocytes, microglia, CD8+ T cells, monocytes and FasL. We could not detect Fas in the brain tissue using Abs available on the market. We found that FasL-expressing CD8+ T cells as well as monocytes localised around HSV-1 infected neurons and other cell types in midbrain and neighbouring cerebellum areas (**Figure 1D**). FasL-positive astrocytes localised more often in HSV-1 infection foci found in cortex and cerebellum (not shown), while FasL-positive microglia were detected in all infection foci (**Figure 1D**). Since the Fas/FasL pathway is involved in apoptosis induction, we decided to determine co-localisation of HSV-1 infection and apoptotic cells (TUNEL+) in brains (**Supplementary Figure 2**). In general, TUNEL-positive cells could be detected within HSV-1 infected sites in midbrain, cerebellum and cortex concomitant with leucocyte infiltration and increased numbers of astrocytes (**Supplementary Figure 2**).

### Reduced CNS Viral Load and Morbidity in HSV-1-Infected Fas- and FasL-Deficient Mice

**Figure 2:**
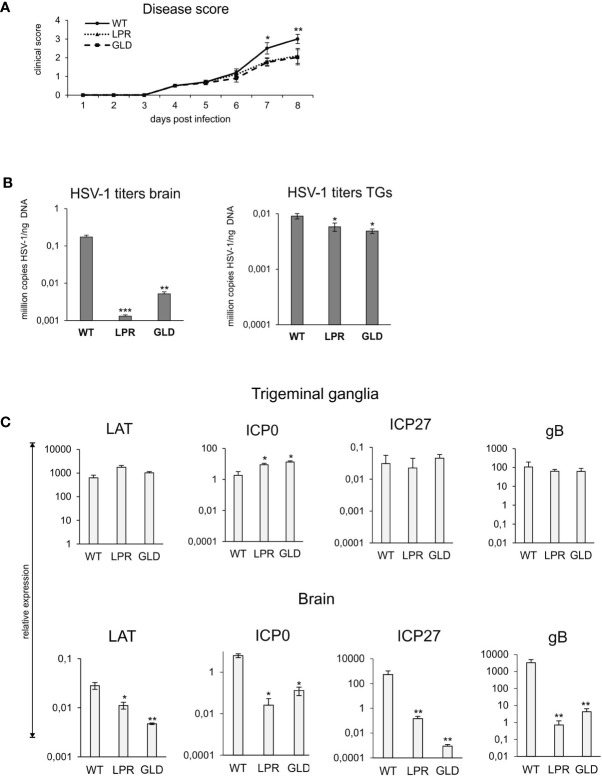
Lack of Fas/FasL decreases morbidity in HSV-1 encephalitis. **(A)** C57BL/6 (WT), B6. MRL-Fas lpr/J (Fas−) and B6Smn.C3-Fasl gld/J (FasL−) mice were infected intranasally and monitored for encephalitis symptoms on a daily basis. **(B)** Viral loads were quantified using qPCR detecting gB gene in DNA extracted from brains and trigeminal ganglia. **(C)** Expression of HSV-1 mRNA for ICP0, ICP27, gB and LAT was measured by qPCR and normalized to beta-actin. Upper panel – trigeminal ganglia, lower panel – brain. All data were presented as mean ± SEM, N = 10. Data analysis was performed by comparing Fas-deficient (lpr) and FasL-deficient (gld) groups with wild-type (C57BL/6) mice. ***indicates P ≤ 0.001, while **P ≤ 0.01 and *P ≤ 0.05.

To directly assess the significance of Fas-FasL interactions in the control of HSV-1 infection, we compared the disease score and viral loads of wild-type and congenic Fas (-) (lpr) and FasL (-) (gld) mice (**Figure 2**). By day 8 after HSV-1 infection, lpr and gld mice showed significantly less clinical symptoms (p ≤ 0.01) (**Figure 2A**) and significantly lower mortality (48% dead animals in WT group *vs.* 21 and 18% dead animals in lpr and gld groups, respectively, p ≤ 0.01). It was followed by significantly lower viral loads in brains (p < 0.001) and trigeminal ganglia (p < 0.05) compared to wild-type (WT) mice (**Figure 2B**).

To assess how the Fas-FasL pathway may influence the lytic/latency cycle in trigeminal ganglia and brains, we compared the expression levels of genes involved in active HSV-1 replication (the immediate early genes ICP0 and ICP27 and the late gB gene) and compared with the expression of LAT, a primary indicator of latency. LATs were detected both in brains and trigeminal ganglia at 8 day p.i., however LATs were more abundant in trigeminal ganglia then in brains (approx. 10^6^ more transcripts). While brains of lpr and gld mice showed significantly less LATs compared to wild-type mice (p ≤ 0.05) (**Figure 2C**), there were no significant differences in LAT expression in trigeminal ganglia (**Figure 2C**). The expression of lytic genes was much higher in brains than in trigeminal ganglia, indicating that latency primarily occurred in trigeminal ganglia of all infected strains (**Figure 2C**). ICP27 and gB transcripts were significantly more abundant in brains of wild-type mice compared to lpr and gld mice (p ≤ 0.01) (**Figure 2C**), in accordance with the higher viral loads in WT mice (**Figure 2B**).

### Immune Cell CNS Infiltration in HSV-1-Infected Fas- and FasL-Deficient Mice

**Figure 3:**
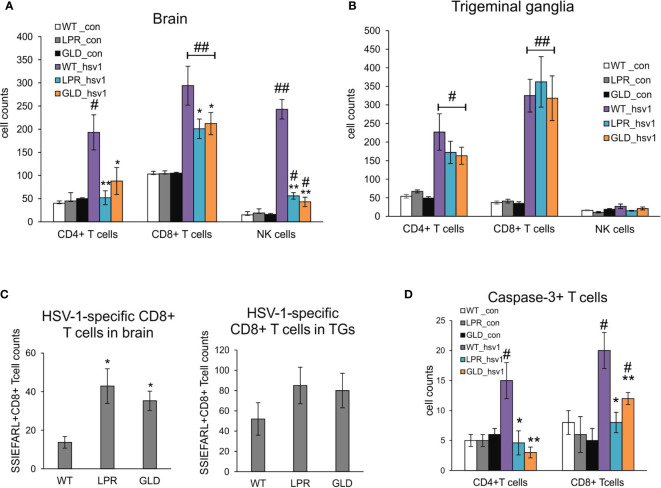
Fas/FasL influences infiltration of T cells and NK cells into the HSV-1 infected brains but not trigeminal ganglia. Cell counts for CD4+ T cells, CD8+ T cells and NK cells were measured by flow cytometry in brains **(A)** and trigeminal ganglia **(B)** of mice uninfected and infected with HSV-1 at 8 d p.i. Data were presented as mean ± SEM, N = 8. Data analysis was performed by comparing Fas-deficient (lpr) and FasL-deficient (gld) groups with wild-type (C57BL/6) mice. **p ≤ 0.001, *p ≤ 0.05. **(C)** HSV-1 specific CD8+ T cells (SSIEFARL+) in brains and ganglia of wild-type, lpr and gld mice measured by flow cytometry. Data were presented as mean ± SEM, N = 8. Data analysis was performed by comparing lpr and gld groups with wild-type (C57BL/6) mice. P ≤ 0.05. **(D)** Numbers of T cells (CD4+ and CD8+) positive for active form of caspase-3 in brains of wild-type, lpr and gld mice at 8 dp.i. Data were presented as mean ± SEM, N = 8. Data analysis was performed by comparing lpr and gld groups with wild-type (C57BL/6) mice and between control and uninfected groups. **indicates P ≤ 0.001, while *P ≤ 0.05 compared to wild-type mice; ^#^indicates P ≤ 0.001, ^##^indicates P < 0.05, in pairs control – infected.

Based on the decreased viral burdens and milder clinical outcome of HSV-1 infection in lpr and gld mice, we hypothesized that lack of Fas or FasL contributed to a better anti-viral response of HSV-1 infection in the brain and trigeminal ganglia. Flow cytometry analysis of CD4+ T cells, CD8+ T cells and NK cells showed significantly lower numbers of T cells and NK cells in brains of lpr and gld mice at 8 d p.i. (p ≤ 0.05) (**Figure 3A**), compared to wild-type mice. No differences in numbers of CD4+ T cells, CD8+ T cells and NK cells were found for trigeminal ganglia (p ≥ 0.05) (**Figure 3B**). Surprisingly, the numbers of HSV-1-specific cytotoxic T cells (CD8+/SSIEFARL+) were significantly increased in brains of lpr and gld mice (p ≤ 0.05) (**Figure 3C**) but not in trigeminal ganglia of all tested strains (p ≥ 0.05) (**Figure 3C**). Furthermore, we detected significantly less CD4+ and CD8+ T cells positive for active caspase-3 form in brains of lpr and gld mice in comparison to wild type mice (p ≤ 0.05) (**Figure 3D**).

**Figure 4:**
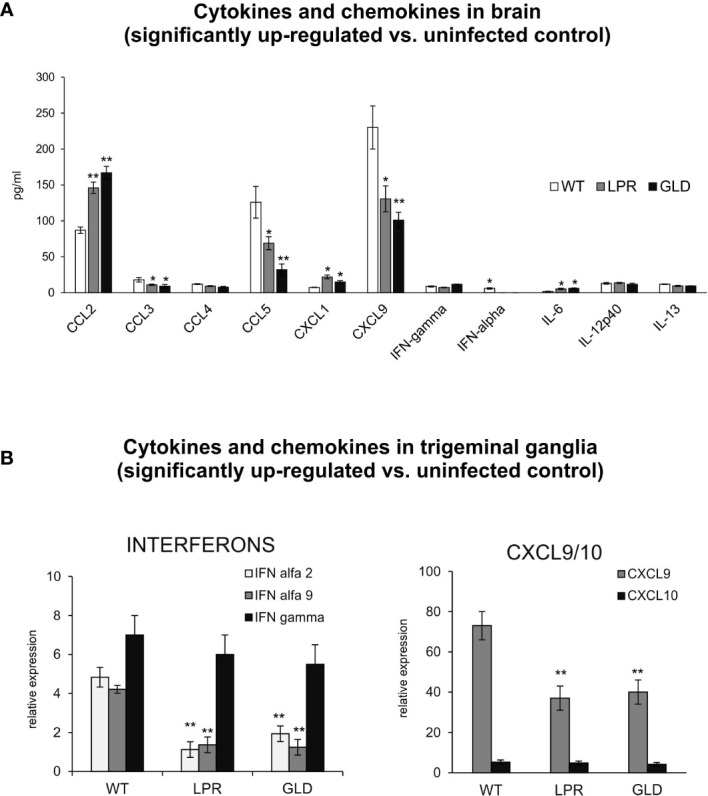
Fas/FasL regulates production of cytokines and chemokines in the HSV-1 infected brain tissue and trigeminal ganglia. **(A)** Chemokines (CCL2, CCL3, CCL4, CCL5, CXCL1, CXCL9) and cytokines (IFN-α, IFN-γ, IL-6, Il-12p40 and IL-13) in the brain tissue at 8 d p.i. were analyzed by Luminex. Data were presented as mean ± SEM, N = 4. Data analysis was performed by comparing Fas-deficient (lpr) and FasL-deficient (gld) groups with wild-type (C57BL/6) mice. **p ≤ 0.001, *p ≤ 0.05. **(B)** Interferons (IFN-α4, IFN-α9 and IFN-γ) and chemokines (CXCL9 and CXCL10) in trigeminal ganglia of wild-type, lpr and gld mice at 8 d p.i. were measured by quantitative real-time PCR. Data were present in mean ± SEM, N = 4. **indicates P ≤ 0.001, while *P ≤ 0.05.

IFNs and other cytokines/chemokines contribute to viral immunity in the CNS (25). Therefore, we evaluated the expression of 23 cytokines and chemokines by Luminex assay. Brains of wild-type mice contained significantly higher levels of CCL3, CCL5, CXCL9, IFN-α compared to lpr and gld mice (p ≤ 0.05) (**Figure 4A**). On the other hand, brains of lpr and gld mice showed significantly higher levels of CCL2, IL-6 and CXCL1/2 compared to wild-type mice (p ≤ 0.05) (**Figure 4A**). No differences among mice strains were detected for CCL4, IL-12 p40, IL-13 and IFN-γ (**Figure 4A**). In trigeminal ganglia, lpr and gld mice demonstrated significantly lower expression of IFN-α 2 and 9 (p ≤ 0.05) (**Figure 4B**). Similarly to brains, CXCL9 expression was significantly decreased in trigeminal ganglia isolated from lpr and gld mice (p ≤ 0.05) (**Figure 4A**), while no differences were detected for IFN-γ and CXCL10.

### Increased Activation of Monocytes and Microglia in Fas/FasL-Deficient Mice

**Figure 5:**
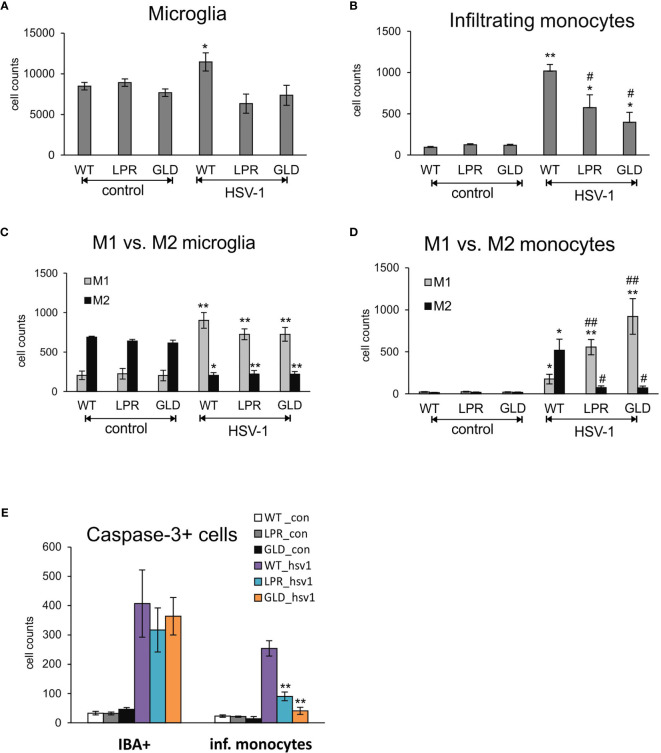
Fas/FasL pathway regulated activation of microglia/monocytes in HSV-1 infected brains. **(A)** microglia cell counts; **(B)** monocyte cell counts in wild-type (C57BL/6), Fas-deficient (lpr) and FasL-deficient (gld) mice at 8 d p.i. measured by flow cytometry in brain tissue homogenates. M1/M2 phenotype of microglia **(C)** and infiltrating monocytes **(D)** in the homogenates prepared as above. **(E)** Numbers of microglia cells (IBA-1+) and infiltrating monocytes positive for active form of caspase-3 in brains of wild-type, lpr and gld mice at 8 dp.i. Data were presented as mean ± SEM, N = 7. Data analysis was performed by comparing Fas-deficient (lpr) and FasL-deficient (gld) groups with wild-type (C57BL/6) mice. **p ≤ 0.001, *p ≤ 0.05, ^#^compared to uninfected control, while ^##^indicates p ≤ 0.01 and ^#^p ≤ 0.05 compared to infected wild-type mice.

Resident microglia and infiltrating monocytes contribute to brain inflammation in HSV-1 infection (26). In our study, we evaluated by flow cytometry whether Fas-FasL signalling can affect the numbers of microglia and infiltrating monocytes (**Figures 5A, B**). The numbers of both microglia (**Figure 5A**) and infiltrating monocytes (**Figure 5B**) in the brain of wild-type mice were significantly increased at day 8 p.i., (p ≤ 0.05). The numbers of microglia in HSV-1 infected lpr and gld mice did however not increase upon infection (**Figure 5A**), while infiltrating monocytes showed significantly higher numbers in Fas- and FasL-deficient mice (p ≤ 0.05) in comparison to uninfected controls (**Figure 5B**). However, the numbers of infiltrating monocytes in HSV-1-infected lpr and gld mice were significantly lower (p ≤ 0.05) in comparison to HSV-1 infected wild-type mice (**Figure 5B**). Monocytes as well as microglia exhibit various types of activated phenotypes, referred to as classical activation phenotypes (M1) typically releasing pro-inflammatory mediators, and alternative activation phenotypes (M2), which possess anti-inflammatory properties (27). Evaluation of microglia phenotypes by flow cytometry showed that all infected strains showed significantly higher numbers of microglia with M1 phenotype and significantly decreased numbers of microglia with M2 phenotype (p ≤ 0.05) (**Figure 5C**). Furthermore, we found significantly more M2 monocytes than M1 monocytes in the brains of HSV-1 infected mice (p = 0.008) (**Figure 5D**), although M1 phenotypes were increased upon infection in all tested strains (p ≤ 0.05) (**Figure 5D**). On the contrary, lpr and gld mice demonstrated significantly more M1 than M2 monocytes (p ≤ 0.05) in comparison to infected wild-type mice (p ≤ 0.01) (**Figure 5D**). Interestingly, while lack of the Fas-FasL pathway had no influence upon caspase-3-dependent apoptosis during HSV-1 infection in all tested strains, it significantly protected infiltrating monocytes from apoptosis (p ≤ 0.01) (**Figure 5E**).

### The Fas/FasL Pathway Regulates Non-Apoptotic Inflammatory Reactions by Microglia

**Figure 6:**
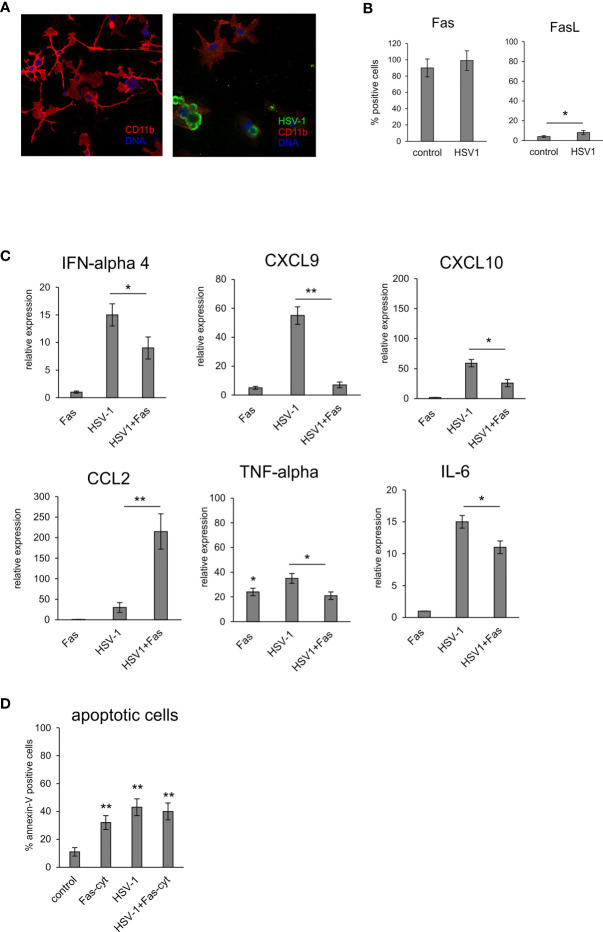
Fas-stimulated microglia in HSV-1 infected cultures down-regulate expression of pro-inflammatory and antiviral cytokines. **(A)** Confocal microphotographs of uninfected (left) and HSV-1 infected (right) microglial cultures at 24 h p.i.; Co-immunofluorescent staining for HSV-1 antigens (green), IBA-1 positive microglia (red). Nuclei were counterstained with DAPI (blue). Magnification x 200. **(B)** Fas and FasL expression on microglial cultures, uninfected and 24 h p.i. measured by flow cytometry. Each bar represents the mean from 3 experiments (N = 3) ± S.E.M., *represents significant differences with p ≤ 0.05. **(C)** Expression of IFN-α 4, CXCL9, CXCL10, TNF-α, IL-6 and CCL2 mRNA at 24 h p.i. in microglia cultures prepared from wild-type (C57BL/6) mice, Fas receptor was stimulated with recombinant mouse FasL. Data were presented as mean ± SEM, N = 4. Data analysis was performed by comparing FasL treated and untreated infected cultures. **p ≤ 0.001, *p ≤ 0.05. **(D)** apoptotic cells (annexin V-positive) in microglia cultures prepared from wild-type (C57BL/6) mice, Fas-dependent apoptosis was induced using recombinant mouse FasL. Each bar represents the mean from 3 experiments (N = 3) ± S.E.M., **represents significant differences with p ≤ 0.001 compared to uninfected control.

To determine how the Fas/FasL pathway modulates inflammatory responses of microglia during HSV-1 infection, we used an *in vitro* model of primary microglia (**Figure 6A**). After 24 hours of HSV-1 infection, primary microglia cultures showed low but a significant increase of FasL expression but not Fas compared to uninfected controls (p = 0.041) (**Figure 6B**). To determine how the Fas/FasL pathway influences production of anti-viral and inflammatory cytokines, we used the recombinant mouse FasL. Addition of FasL to uninfected microglia cultures led to a significant up-regulation of TNF-α expression (p = 0.49) (**Figure 6C**). Upon HSV-1 infection, microglia cultures significantly up-regulated their expression of IFN-α4, CXCL9, CXCL10, TNF-α and IL-6 mRNA (p ≤ 0.05) (**Figure 6C**), while addition of recombinant FasL led to significant down-regulation of the observed effects (p ≤ 0.05) (**Figure 6C**). An opposite effect was observed for CCL2; stimulation of Fas significantly up-regulated CCL2 expression compared to infected cultures (p = 0.008) (**Figure 6C**). In order to dissect whether the observed effect was apoptosis-related, we tested for apoptosis upon activation of Fas receptor by recombinant FasL in uninfected and infected microglia cultures (**Figure 6D**). While FasL-treated uninfected cultures and HSV-1 infected cultures showed significant induction of apoptosis (p ≤ 0.05) (**Figure 6D**), stimulation of Fas receptor in HSV-1 cultures did not increase apoptosis in HSV-1 infected cultures (**Figure 6D**).

**Figure 7:**
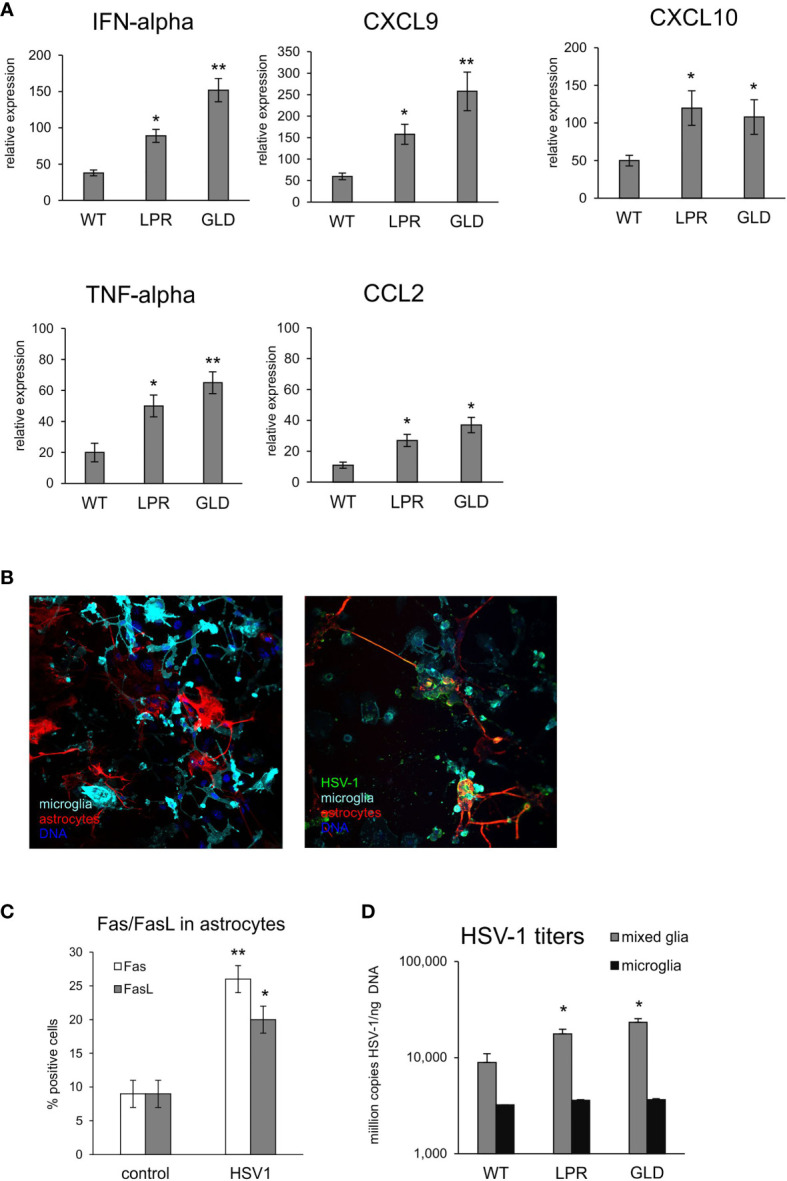
Fas/FasL pathway is involved in microglia activation during HSV-1 infection *in vitro*. **(A)** Expression of IFN-α4, CXCL9, CXCL10, TNF-α and CCL2 mRNA at 24 h p.i. in microglia cultures prepared from wild-type (C57BL/6), Fas-deficient (lpr) and FasL-deficient (gld) mice. Data were presented as mean ± SEM, N = 4. Data analysis was performed by comparing lpr and gld groups with wild-type mice. **p ≤ 0.001, *p ≤ 0.05. **(B)** Confocal microphotographs of uninfected (left) and HSV-1 infected (right) mixed glial cultures at 24 h p.i.; Co-immunofluorescent staining for HSV-1 antigens (green), GFAP+ astrocytes (red), IBA-1 positive microglia (red). Nuclei were counterstained with DAPI (blue). Magnification x 200. **(C)** Fas and FasL expression measured by flow cytometry on uninfected and 24 h p.i. astrocytes from mixed glial cultures prepared from wild-type mice. Each bar represents the mean from 3 experiments (N = 3) ± S.E.M., *represents significant differences with p ≤ 0.05, while ** with p ≤ 0.01. **(D)** HSV-1 titers in DNA extracted from 24h infected wild-type mixed glia and purified microglia cultures using qPCR detecting gB gene. Each bar represents the mean from 4 experiments (N = 4) ± S.E.M., *represents significant differences with p ≤ 0.05, while ** with p ≤ 0.01.

To further determine how the Fas/FasL pathway may regulate active phenotype of microglia, we prepared primary microglia cultures from wild-type, lpr and gld mice (**Supplementary Figure 3**). Microglia prepared from all tested strains showed no differences in percentage of M1 and M2 phenotype, with significantly more M2 phenotype cells (p ≤ 0.05) (**Supplementary Figure 3**). Infection with HSV-1 led to significant increase of M1 phenotype, and a decrease in M2 phenotype (p ≤ 0.05) (**Supplementary Figure 3**). HSV-1 infected microglia from lpr and gld mice demonstrated significantly more M1 phenotype cells compared to microglia from wild-type mice (p ≤ 0.05) and significantly more M1 cells to M2 cells (p ≤ 0.05) (**Supplementary Figure 3**). Stimulation of microglia prepared from all tested strains with poly I:C led to significant increase of M1 phenotype (p ≤ 0.05), albeit without strain differences. Furthermore, we also tested for expression of cytokines and chemokines in HSV-1 infected microglia cultures prepared from wild-type, lpr and gld mice (**Figure 7A**). HSV-1 infected Fas- and FasL-deficient microglia produced significantly more IFN-α4, CXCL9, CXCL10, TNF-α and CCL-2 than infected wild-type microglia (p ≤ 0.05) (**Figure 7A**). To elucidate if astrocytes can be contribute to Fas/FasL pathway in infected brains, we prepared mixed glial cultures, consisting approximately of 50% microglia and 50% glial cells (**Figure 7B**) and stained for Fas and FasL (**Figure 7C**). HSV-1-infected astrocytes significantly (p ≤ 0.05) up-regulated both Fas and FasL expression, although the general expression was low (**Figure 7C**). In mixed glial cultures we detected no differences in the viral loads for microglia, while astrocytes from Fas- and FasL-deficient mixed glial cultures showed increased viral loads in comparison to infected wild-type cultures (p ≤ 0.05) (**Figure 7D**).

## Discussion

Our present study demonstrates the role of Fas/FasL in the HSV-1-infected brain; the cell death receptor pathway protects from virus infection by inducing an inflammatory response within the CNS, but at the same time the virus appears to benefit from the Fas/FasL pathway, which facilitates its persistence and shedding throughout the CNS.

The Fas/FasL pathway is important for immune homeostasis and the elimination of infected cells *via* apoptotic signaling pathways (28). Here, we found that during HSV-1 CNS infection, up-regulation of Fas and FasL expression correlated in a space- and time-dependent manner with HSV-1 infection. Fas and FasL were detected in most areas of the brain. The brain regions with highest viral loads (thalamus, hypothalamus, midbrain and brain stem) demonstrated an early increase in Fas mRNA and a delayed increase in FasL mRNA expression (48h of difference). We did not detect Fas or FasL expression on HSV-1-infected or neighboring neurons, nor on any apoptotic neurons. We can therefore conclude that Fas/FasL pathway is not the direct pathway through which HSV-1-infected neurons are eliminated. This contrasts to the reported up-regulation of Fas on infected neurons during brain infection with reovirus (29), West Nile virus (13), Chandipura virus (30) and murine hepatitis virus (14). For these brain infections, up-regulation of Fas seems necessary to eliminate infected neuronal cells *via* infiltrating T cells or in an autocrine manner. For FasL, Baloul et al. showed *in vivo* that a pathogenic strain of rabies virus triggers the early upregulation of FasL on neurons and non-neuronal glial cells, which leads to apoptosis of infiltrating T cells and thus a reduced killing of virus-infected cells (16). As shown here, microglia and astrocytes within infected sites together with infiltrating monocytes and T cells are the main sources of Fas-FasL interactions within the HSV-1 infected brain. Furthermore, the data from the brains of HSV-1-infected mice contrast to what we have previously observed in non-neuronal tissues during HSV-2 infection where both Fas and FasL expression are enhanced on infected epithelial cells, rendering them apoptosis-resistant (17).

The mechanisms through which HSV-infected epithelial and glial but not neuronal cells up-regulate Fas and FasL are unknown and many different inductive pathways are involved. In the CNS, it has been shown that an injury within the spinal cord leads to Fas-dependent infiltration of macrophages and neutrophils causing neuronal death (31). Infiltrating cells bear FasL receptor, which in turns induces microglia and astrocyte activation and can further attract immune competent cells (32, 33). The ability of HSV-1 to successfully evade immune detection is critical to establish lifelong latency in neurons. After infectious HSV is cleared, the latently infected neurons provide the only reservoir of virus for future reactivation (34). The host immune response plays an important role in limiting viral replication and spread following reactivation (34). Therefore, we can conclude that HSV-1 infected neurons do not express Fas or FasL which obviously is of advantage for the virus, since Fas-expressing neurons would become sensitive to apoptosis induced by infiltrating T cells or monocytes. At the same time, FasL-expressing neurons could have induce apoptosis in infiltrating immune competent cells. Apoptosis of immune competent cells is associated with production of cytokines/chemokines and helps to present viral antigens by phagocytosis of dying cells (35). We could detect abundant FasL expression only within sites of excessive inflammation, where FasL-bearing monocytes and CD8+ T cells but also microglia were identified.

HSV-1 can differentially modulate apoptosis in immune and non-immune cells, which may favor interference with the host antiviral immune response while allowing viral replication in neurons (36, 37). During acute brain infection, sensitivity to apoptosis in HSV-1 infected cells can be also different, depending on the localization. For example, while ependymal cells were shown to be highly sensitive to HSV-induced apoptosis, neuronal and ganglia cells were undergoing moderate or no apoptosis during *in vivo* infection (38). Taking into account the fact that infiltrating T cells and monocytes can limit the viral spread, it is not surprising that HSV encodes proteins which were shown to inhibit this pathway (37). For example, the RNR domain of HSV R1 can directly bind to the caspase-8 death effector domain and prevents caspase-8 activation, leading to suppression of extrinsic apoptotic signaling (39). Furthermore, LAT, virus transcript expressed by latently infected neurons, can also inhibit caspase-8 and caspase-9-induced apoptosis, leading to inhibition of CD8+ T cell-killing of latently infected neurons (40). Cymerys et al. showed that upon HSV-1 infection *in vitro*, both microglia and astrocytes are resistant to Fas-induced apoptosis (41).

Our previous studies demonstrated increased viral loads and mortality during HSV-2 infection in Fas- and FasL-deficient mice, resulting from uncontrolled inflammation and an impeded local NK and T cell response (17). Monocytes and neutrophils accumulated within the HSV-2-infected epithelium of Fas- and FasL-deficient mice, leading to uncontrolled inflammation and destruction of the tissue. Krzyzowska et al. have previously shown that Fas/FasL dependent apoptotic pathway was not only a crucial mechanism for elimination of the inflammatory cells present in the HSV-2 infected sites, but also helped to develop the local chemokine and cytokine milieu, necessary for mounting proper anti-viral response (17, 22). Similarly, HSV-1 infected BALB/c and C57BL/6 mice bearing mutations in Fas (lpr) and FasL (gld) displayed more severe herpetic stromal keratitis (HSK) compared to wild-type mice. It was further demonstrated that increased disease was due to lack of Fas expression on infiltrating monocytes and neutrophiles (42). Therefore, we expected that lack of Fas- and FasL in HSV-1 infection would led to more severe encephalitis and mortality in mice. Surprisingly, Fas- and FasL-deficient (lpr and gld) mice infected with HSV-1 showed reduced morbidity and mortality compared to wild-type mice as a result of significantly lower virus replication in the brains and trigeminal ganglia of Fas- and FasL-deficient mice. These results are in accordance with other studies: In a murine model of flavivirus-induced encephalitis, mice deficient in either the granule exocytosis- or Fas-mediated pathway of cytotoxicity showed delayed and reduced mortality when infected with Murray Valley encephalitis virus (MVE) (43). In mice lacking FasL (gld), infection with the neuroinvasive rabies virus strain was less severe, and the number of CD3 T cells undergoing apoptosis was smaller than that in normal mice (16). Here, while no difference in the numbers of T cells and NK cells were found for trigeminal ganglia, HSV-1 infected brains contained significantly fewer T cells and NKs. Furthermore, as shown previously by Baloul et al. (16), the lower levels of apoptotic T cells (both CD4+ and CD8+) in mice lacking Fas or FasL strongly supports the notion that T cells infiltrating HSV1-infected CNS undergo apoptosis *via* Fas/FasL pathway. Additionally, lack of Fas or FasL expression did not impede migration of HSV-1 specific cytotoxic CD8+ T cells into the brains, it actually led to better infiltration of HSV-1 specific cytotoxic T cells. Similar results were observed in CNS infection with murine hepatitis virus, where no differences were found in the frequency of virus-specific CD8+ T cells infiltrating the CNS of both infected Fas-deficient and wt mice (14). Thus, Fas and FasL deficiency appear to reduce the viral load in many different viral infections.

Our data show that the Fas/FasL pathway leads to excessive neuroinflammation during HSV-1 infection, which is associated with a diminished anti-viral response. Normally, HSV-1 CNS infection induces cytokines and chemokines that contribute to the control of HSE, e.g. type I IFN, IFN-γ, IL-1β and IL-6 (25, 44–47). At the same time, both type I IFN and IL-1β contribute to immunopathology, with the brain being a particularly sensitive organ to long-term effects of type I IFN and inflammation (47). Here, we found that the Fas/FasL pathway had no influence upon IFN-γ levels in brains and trigeminal ganglia in HSV-1 infected mice, while IFN-α was decreased in Fas and FasL-deficient mice. Interleukin 6 is another prominent pro-inflammatory molecule that confers resistance to primary HSV-1 infection *via* a STAT3-dependent pathway (45, 46). We found that mice without a functional Fas/FasL pathway had actually increased levels of IL-6 in the CNS, which could contribute to better survival of lpr and gld mice. CXCL9, CXCL10 and CXCL11 chemokines attract T cells and NK cells to infiltrate infected sites (48, 49). Previously, we showed that monocytes from HSV-2 infected Fas- and FasL-deficient mice underwent delayed apoptosis and produced significantly less CXCL9, CXCL10 and TNF-α than monocytes in the wild type mice (17). Similarly, in this study we found that brains of Fas- and FasL-deficient mice showed significantly lower levels of CCL3, CCL4, CCL5 and CXCL9 compared to wild-type mice. During rabies infection of brain, Fas-deficient mice also show decreased levels of CCL2, IL-6, TNF-α, IL-1β (16). In contrast to our previous studies with HSV-2 in genital infection, lack of functional Fas/FasL pathway led to increased expression of CCL2 and CXCL1/2 in brains. As shown by Conrady et al., abundant production of CXCL1/2, CCL2 and CCL5 can induce the recruitment of peripheral immune cells that in turn may contribute to amplify the global inflammation triggered by the virally-infected cells (50). Thus, the Fas/FasL pathway has a strong impact on cytokine and chemokine production in various viral infections but differ between anatomical sites and also to a certain degree in different infections.

In neuroinflammatory conditions, microglia and infiltrating monocytes are thought to contribute to pathogenicity (50), however, in the case of viral encephalitis, it remains unclear whether microglia are beneficial or detrimental to disease outcomes, or possibly both. Under physiological conditions, microglia are in a resting state; however, upon exposure to infectious and traumatic stimuli, microglia produce various substances such as reactive oxygen species (ROS), nitric oxide, pro-inflammatory cytokines chemokines, which contribute to the clearance of pathogenic infections (51, 52). Upon HSV-1 infection, microglial cells undergo an abortive infection and induce a burst of pro-inflammatory cytokine and chemokine production. It was shown that NO produced by microglial cells helps to reduce viral replication in the infected neurons but not in astrocytes, it also down-regulates cytokines important for anti-viral response in the neuronal microenvironment (41). Therefore, prolonged or strong local production of NO may further add to HSV-1 local spread and further pathology by limiting the specific anti-viral immune response. However, Uyar et al., showed that an early (6 day post infection) microglial response followed by sustained infiltration of monocytes and T cells into the brain as the key components for a better recovery from HSE (26). The pharmacological depletion of microglia increased brain viral loads and mortality in mouse models of West Nile virus encephalitis and mouse hepatitis virus encephalitis, suggesting that the initiation of the innate and adaptive immune responses requires functional microglia (53, 54). Upon HSV-1 infection, microglial cells undergo an abortive infection and induce a burst of pro-inflammatory cytokine and chemokine production. In HSV-1 infected lpr and gld mice we found high levels of infiltrating monocytes in the CNS, suggesting that these cells can be cleared *via* a Fas/FasL-dependent pathway. We found no difference in the numbers of microglia in Fas/FasL-deficient animals which is in line with the fact that microglia are resistant to Fas/FasL-mediated apoptosis (41, 55).

Classically, macrophages and microglia have been defined as being polarized, meaning they express two major activation states: pro-inflammatory M1 and anti-inflammatory M2 (27). The ability to shift phenotypes allows microglia to maintain homeostasis within the CNS. Fas/FasL deficiency promoted the maturation of M1 macrophages which might explain why gld and lpr mice had a better outcome of the infection. It is known that M1 macrophages are refractory to HSV-1 replication, at least *in vitro* (56), while M2 macrophages promote viral replication (57). Activated microglia can control virus replication in the brain by producing inflammatory cytokines and chemokines such as IL-6, IL- 1β, type I interferons (IFN-I), CXCL-10, CCL2 and CCL5, which our data partly confirm. However, overactive immune responses may contribute to the long-term neuropathological sequelae associated with HSE. Our data demonstrate that lack of Fas/FasL leads to increased numbers of M1 activated monocytes in the HSV-1-infected brains, while it has a moderate effect upon M1 activation of microglia.

Of note, while *in vitro* stimulation of the Fas receptor on microglia using an activating antibody induced only moderate inflammatory response and apoptosis, HSV-1 infected microglia were up-regulating their production of pro-inflammatory and anti-viral cytokines and chemokines such as CCL2, CXCL9, CXCL10, IFN-α, TNF-α and IL-6. However, HSV-1-infected microglia stimulated through Fas were both resistant to Fas-mediated apoptosis and were down-regulating inflammatory response. HSV-1 infected microglia from Fas- and FasL-deficient mice showed much more efficient inflammatory responses in comparison to microglia from wild type mice which confirms the role of Fas/FasL in modulating inflammatory responses. *Thus, we propose that HSV-1 modulates Fas-mediated pro-inflammatory pathways within the CNS in a manner which disturbs Fas-mediated apoptotic and proinflammatory response upon migration of FasL bearing lymphocytes into the infected site.*

Based on our data, we propose that upon HSV-1 infection, CNS-resident cells produce cytokines, which attract FasL-positive peripheral immune cells such as monocytes and T cells. This leads to amplification of the chemotactic gradient and, thereby, to further infiltration of leukocytes. Infiltrating virus-specific cytotoxic T cells can kill the infected neurons and astrocytes, while FasL-bearing monocytes will amplify the chemotactic gradient. However, since HSV-1 infected cells are both resistant to Fas-induced apoptosis and down-regulate non-apoptotic inflammatory signaling, it can paradoxically lead to excessive local inflammation that contributes to increased morbidity and mortality.

We conclude that within the CNS, Fas/FasL signaling seems to take part in the complex regulation of the local inflammatory reaction, which is corroborated by HSV-1, leading to further disturbances in antiviral immunity as well as to an excessive neuroinflammation.

## Data Availability Statement

The raw data supporting the conclusions of this article will be made available by the authors, without undue reservation.

## Ethics Statement

The animal study was reviewed and approved by Animal Research Ethical Committee of Gothenburg.

## Author Contributions

Conception and design: MK and KE. Analysis and interpretation: MK, AK, KS and KT. Drafting the manuscript: MK and KE. All authors contributed to the article and approved the submitted version.

## Funding

This work was funded by the National Science Centre Poland (grant numbers 2015/18/M/NZ6/00414 and 2020/37/B/NZ6/03284), the Swedish state under the agreement between the Swedish government and the county councils, the ALF-agreement (ALFGBG-827291), the Swedish Research Council (2020-02732), Rune and Ulla Amlövs stiftelse 2020 for KE, Wenner-Gren Foundation scholarship no. GFoh2019-0012 and More2020 individual scholarship from Vastra Götalandsregionen 2018 for MK.

## Conflict of Interest

The authors declare that the research was conducted in the absence of any commercial or financial relationships that could be construed as a potential conflict of interest.

## Publisher’s Note

All claims expressed in this article are solely those of the authors and do not necessarily represent those of their affiliated organizations, or those of the publisher, the editors and the reviewers. Any product that may be evaluated in this article, or claim that may be made by its manufacturer, is not guaranteed or endorsed by the publisher.

## Supplementary Material

Supplementary Figure 1 Presence of HSV-1 in different brain parts at 6-8 days post infection of C57BL/6 mice. **(A)** Viral loads were quantified using qPCR detecting gB gene in DNA extracted from different brain parts (CH – cerebral hemispheres, diencephalon – midbrain, brainstem and medulla), cerebellum and olfactory bulb. N = 10 animals at each time point. Data were presented as mean ± SEM. **(B)** Representative confocal microphotographs of HSV-1 in different brain parts at 8 d p.i. Co-immunofluorescent staining for HSV-1 antigens (green), GFAP+ astrocytes (red), IBA-1 positive microglia and NeuN-positive neuronal cells (red or turquoise) and myelin basic protein (turquoise). Nuclei (blue) were counterstained with DAPI. Magnification x 200.

Supplementary Figure 2 Expression of FasL and apoptosis in different brain parts of C57BL/6 mice at 8 day post HSV-1 infection. **(A)** Representative confocal microphotographs of co-immunofluorescent staining for HSV-1 antigens (green), GFAP+ astrocytes (red), FasL (turquoise) in (from left to right) ependyma, cortex and midbrain. Nuclei (blue) were counterstained with DAPI. **(B)** Representative confocal microphotographs of co-immunofluorescent staining for HSV-1 antigens (green), apoptotic cells (TUNEL+, red) and astrocytes (GFAP+, turquoise), microglia (IBA-1+, turquoise) and CD8+ T cells (turquoise) in ependyma, midbrain and brain stem.

Supplementary Figure 3 Fas/FasL pathway in microglia M1/M2 phenotype. Microglia cultures prepared from wild-type (C57BL/6), Fas-deficient (lpr) and FasL-deficient (gld) neonatal mice were infected with HSV-1 or poli I:C-treated for 24h. M1 cells were identified by flow cytometry as CD86/iNOS-positive, while M2 as CD206/Arg-1-positive. Bars represent mean ± SEM, N = 4. *indicates P < 0.05, compared to wild-type microglia, while ^#^indicates P < 0.05, in pairs M1-M2.
