# Supplementary figures and images for: Fas/FasL Contributes to HSV-1 Brain Infection and Neuroinflammation
Source: Front Immunol. 2021 Aug 30;12:714821. doi: 10.3389/fimmu.2021.714821 (PMC8437342; doi:10.3389/fimmu.2021.714821)

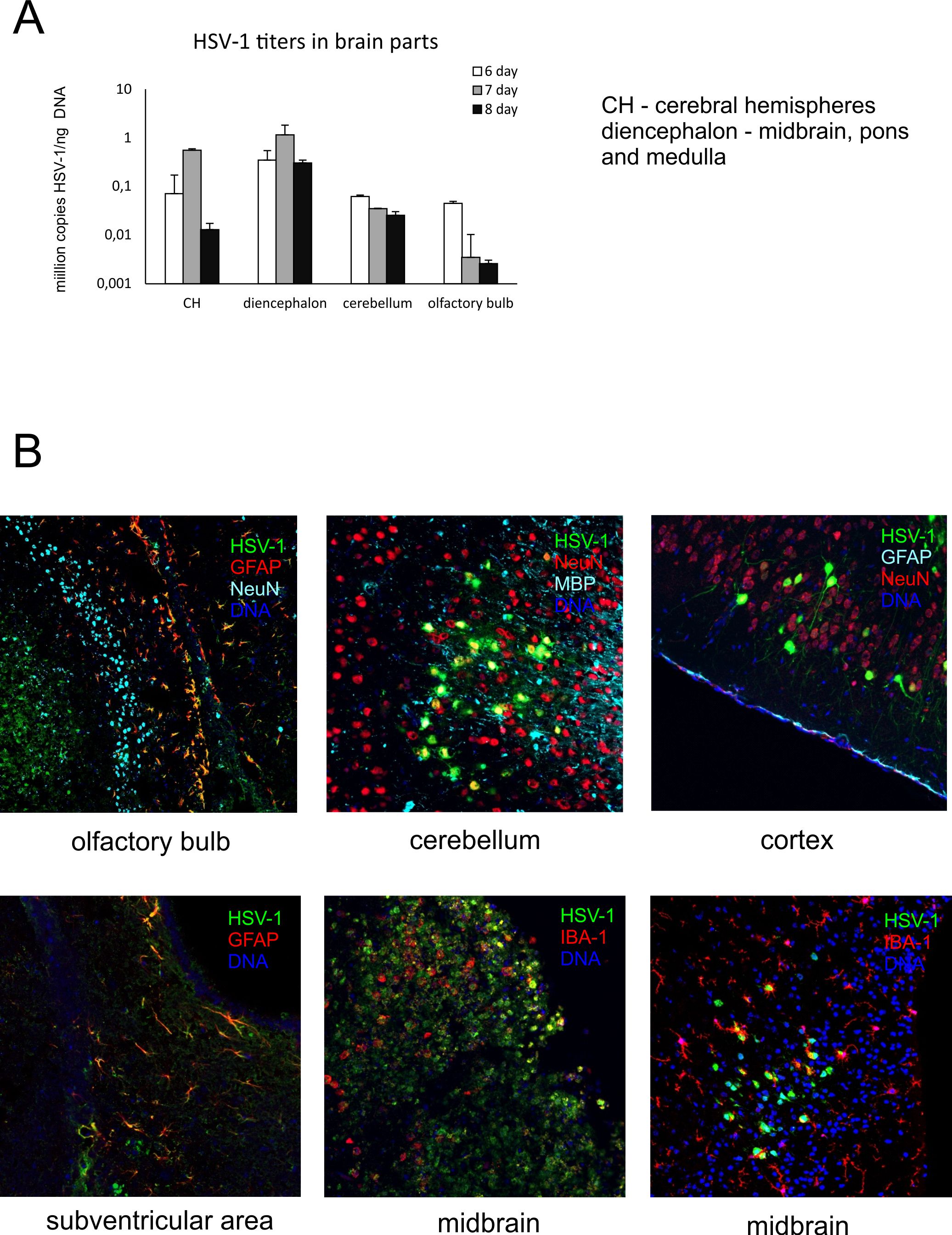

Supplement: Supplementary Figure 1 — Presence of HSV-1 in different brain parts at 6-8 days post infection of C57BL/6 mice. (A) Viral loads were quantified using qPCR detecting gB gene in DNA extracted from different brain parts (CH – cerebral hemispheres, diencephalon – midbrain, brainstem and medulla), cerebellum and olfactory bulb. N = 10 animals at each time point. Data were presented as mean ± SEM. (B) Representative confocal microphotographs of HSV-1 in different brain parts at 8 d p.i. Co-immunofluorescent staining for HSV-1 antigens (green), GFAP+ astrocytes (red), IBA-1 positive microglia and NeuN-positive neuronal cells (red or turquoise) and myelin basic protein (turquoise). Nuclei (blue) were counterstained with DAPI. Magnification x 200. [file Image_1.jpg]

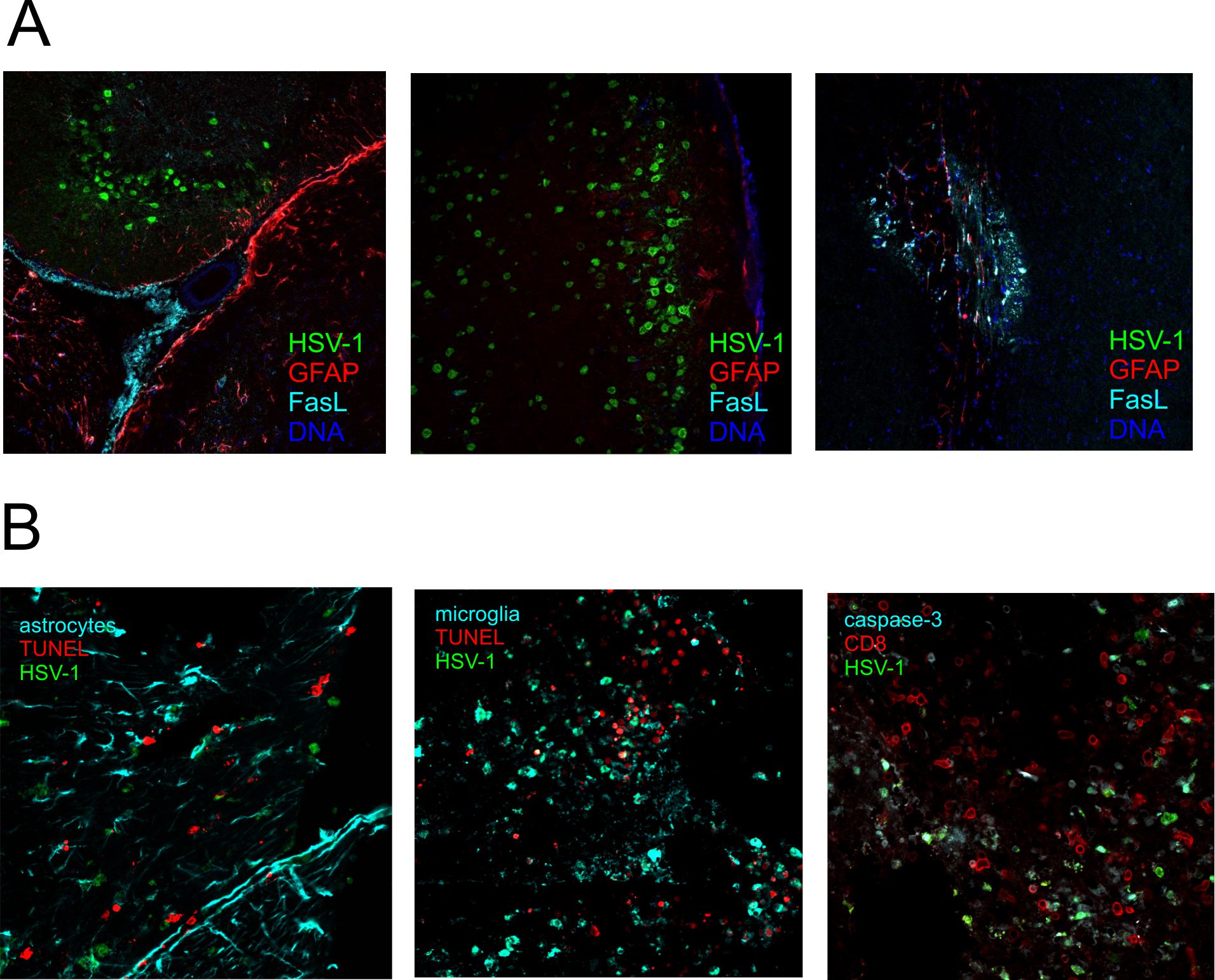

Supplement: Supplementary Figure 2 — Expression of FasL and apoptosis in different brain parts of C57BL/6 mice at 8 day post HSV-1 infection. (A) Representative confocal microphotographs of co-immunofluorescent staining for HSV-1 antigens (green), GFAP+ astrocytes (red), FasL (turquoise) in (from left to right) ependyma, cortex and midbrain. Nuclei (blue) were counterstained with DAPI. (B) Representative confocal microphotographs of co-immunofluorescent staining for HSV-1 antigens (green), apoptotic cells (TUNEL+, red) and astrocytes (GFAP+, turquoise), microglia (IBA-1+, turquoise) and CD8+ T cells (turquoise) in ependyma, midbrain and brain stem. [file Image_2.jpeg]

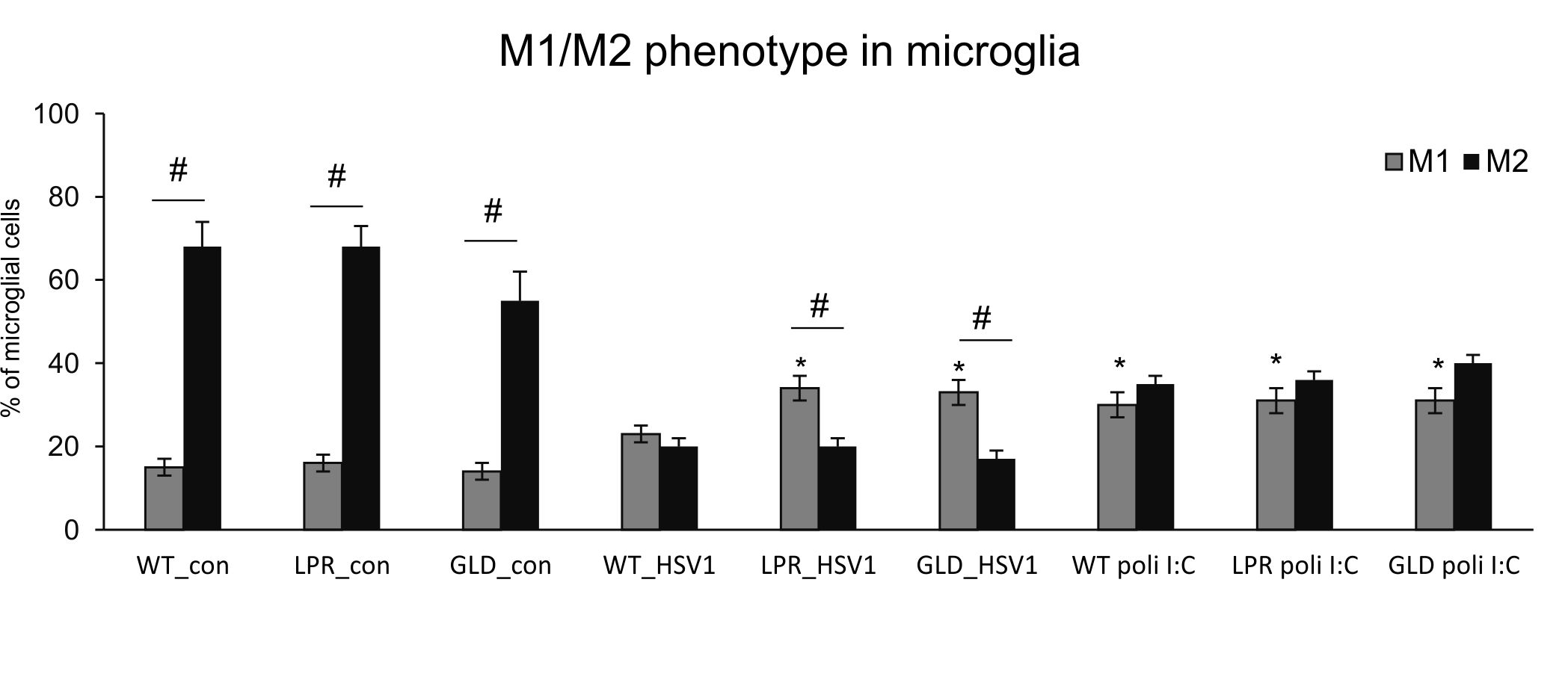

Supplement: Supplementary Figure 3 — Fas/FasL pathway in microglia M1/M2 phenotype. Microglia cultures prepared from wild-type (C57BL/6), Fas-deficient (lpr) and FasL-deficient (gld) neonatal mice were infected with HSV-1 or poli I:C-treated for 24h. M1 cells were identified by flow cytometry as CD86/iNOS-positive, while M2 as CD206/Arg-1-positive. Bars represent mean ± SEM, N = 4. *indicates P < 0.05, compared to wild-type microglia, while #indicates P < 0.05, in pairs M1-M2. [file Image_3.jpeg]
